# Yellow fever outbreak potential in Djibouti, Somalia and Yemen: a mathematical modelling study

**DOI:** 10.1186/s44263-026-00284-9

**Published:** 2026-06-08

**Authors:** Keith Fraser, Laurence Cibrelus, Jennifer Horton, Chiori Kodama, J. Erin Staples, Katy A. M. Gaythorpe

**Affiliations:** 1 MRC Centre for Global Infectious Disease Analysis, School of Public Health, Imperial College London, Wood Lane, London, United Kingdom; 2 World Health Organization, Avenue Appia 20, 1211 Geneva, Switzerland; 3 Division of Vector-borne Diseases, Centers for Disease Control and Prevention, Fort Collins, Colorado, USA

**Keywords:** Yellow fever, Outbreak, Transmission risk, Mathematical modelling

## Abstract

**Background:**

The importation of arbovirus diseases into new countries is a global concern. This risk is exacerbated by human movement and climate change effects. In the World Health Organization (WHO) Eastern Mediterranean regional office, three countries - Djibouti, Somalia, and Yemen - are currently classified as potential or moderate risk for yellow fever (YF) outbreaks.

**Methods:**

Here we present a quantitative assessment of the risk of introduction and propagation of yellow fever virus (YFV) transmission in Djibouti, Somalia and Yemen. This modelling has two components: i) projecting the risk of importation of infectious individuals into the countries of interest using a radiation model of human movement and ii) estimating the risk of onward transmission given importation using a dynamic compartmental model of yellow fever virus transmission. Both components are multiplicatively combined to give an overall relative outbreak risk combining both risk of importation and risk of an outbreak given importation.

**Results:**

We show that areas such as the western coast of Yemen, regions of Somalia bordering Ethiopia and Kenya, and Djibouti City have potential for YF outbreaks (where the estimated probability of an outbreak given an imported infectious case is over 50%). This is due to environmental suitability for transmission based on factors such as temperature and projected human mobility between endemic and at-risk regions.

**Conclusions:**

Countries bordering existing YF endemic regions are potentially vulnerable to both introduction of YF cases and subsequent outbreak spread. This promotes the awareness of YF importation potential when conducting clinical surveillance in at-risk countries.

## Background

Yellow fever (YF) is a viral haemorrhagic fever endemic in tropical regions of Africa and South America with rapid propagation and amplification, particularly in urban settings, and potential for international dissemination [1]. Control is primarily conducted through early identification of cases, vaccination of susceptible people, and vector control activities [2]. With multiple transmission cycles, a sylvatic reservoir in non-human primates (NHPs), and no treatment available, YF eradication is not feasible [1]. Therefore, targets for YF elimination focus on outbreaks, particularly in urban settings. These targets are set by the World Health Organization (WHO) Eliminate Yellow Fever Epidemics (EYE) Strategy [1] which focuses on i) protecting at-risk populations, ii) preventing international spread, and iii) containing outbreaks rapidly. To achieve these aims, a clear categorisation of risk is crucial to tailor public health measures.

The risk of YF can be examined in a range of ways [3, 4]. This may be informed by data on recent outbreaks, current vaccination coverage or the environmental conditions. These factors help to project the transmission intensity (for example through the basic reproduction number or force of infection) or environmental suitability for the vector. The EYE strategy risk analysis working group has worked extensively on defining appropriate criteria to assess risk [1]. However, depending on the metric used, this risk assessment can vary; for example, transmission intensity estimates may be affected by climate change and estimates of under-immunised populations may be affected by recent vaccination activities [5]. Climate change may lead to the establishment of yellow fever virus (YFV) in regions where it is not currently circulating [6]. This is of concern as the population would lack natural or vaccine-induced immunity, creating the potential for large outbreaks. Similarly, human activities such as changing movement patterns, deforestation or changing agricultural practices can lead to changes in virus circulation and human risk [7]. As a result, the risk of YF is in flux.

Assessing risk of YF is critical, particularly in areas bordering existing endemic regions. This is to ensure that interventions and surveillance can be strengthened to capture and respond to YF disease occurrence in new areas and populations. Focusing only on current endemic areas could miss populations at potential risk of future outbreaks. Mathematical modelling is a way of bringing together information on the current epidemiological situation with mechanisms for how that situation may change in future, for example, because of changes in the environment or population.

Here we examine the outbreak potential of YF in the Republic of Djibouti, the Federal Republic of Somalia, and the Republic of Yemen (hereafter referred to as Djibouti, Somalia and Yemen respectively), three countries which border endemic or ‘high-risk’ regions and which are considered to be at moderate or potential risk of YF outbreaks by the WHO EYE Strategy (Additional File 1: Fig S1) [1]. Firstly, we capture the potential spatial spread of an outbreak using estimated values of the subnational force of infection and a radiation model of human movement. Secondly, we use a stochastic, dynamic model of YFV transmission to estimate the risk of a single imported case leading to an outbreak. In this way, we examine the risk that YFV could be imported into a district (administrative level 2) and then the potential for that case to propagate an outbreak once there.

## Methods

### Overview

**Fig. 1:**
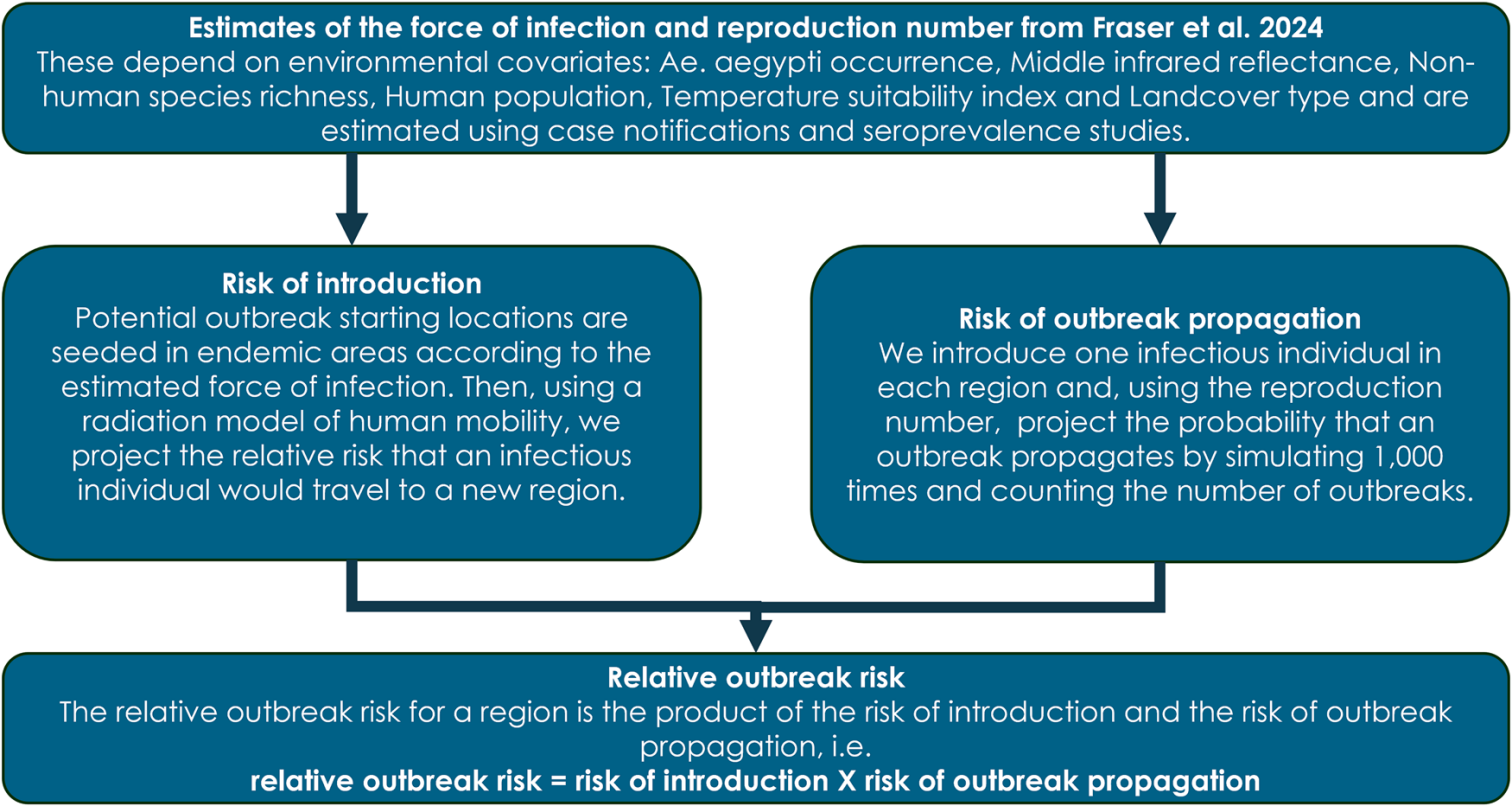
Flowchart of methods from the estimates of the force of infection and reproduction number per region from [11], to the resulting relative outbreak risk

We examine two components of risk: 1) the risk of introduction/importation for Djibouti and Somalia and 2) the risk of a propagated outbreak given introduction for Djibouti, Somalia and Yemen. To examine the risk of introduction, we couple a simple model of human movement, a radiation model, with the known natural history of YFV infection to give a relative risk that an infectious person might travel to a new area from an endemic area. We calculate the risk of a propagated outbreak due to the introduction of an infectious individual using a mechanistic model of YFV transmission. Through the combination of these approaches, we can highlight areas that may be vulnerable to YFV introduction and ongoing transmission. Figure 1 shows the methodological steps which we will expand in the following sections.

### Risk of introduction

The risk of introduction relies on a radiation model of human movement, which depends on the population size and distance between districts [8]. The radiation model is chosen as it performs well at approximating the distribution of long-distance movements [9], has been found to be the most appropriate model of movement in other disease outbreaks such as polio [10], and is parameter free, which means that no estimation is required. The general form is shown below:

1 $${M}_{ij}=\frac{{p}_{i}{p}_{j}}{({p}_{i}+{s}_{ij})({p}_{i}+{p}_{j}+{s}_{ij})},$$

where *p*_*i*_ is the population of district *i* and *s*_*ij*_ is the population in the circle centred on district *i* and touching district *j*[8]. The risk of introduction to district *i* is given by:

2 $${R}_{intr{o},{i}}=\sum _{j}{C}_{j}{M}_{ij}{\psi }_{ij},$$

where *C*_*j*_ is the number of cases in district *j* and *ψ*_*ij*_ is a measure of how porous any country borders between districts *i* and *j* are; we assumed *ψ*_*ij*_ = 1. In this study, we focus on the risk within districts in Djibouti, Somalia, and surrounding countries. Yemen is not included in this component as we assume cross-border movement is land-based through the radiation model.

We examine the potential spread of an outbreak of YF originating in a region in YF endemic countries bordering Somalia and Djibouti, see Additional file 1: Fig. S1 for countries. We sample from potential start points within the countries directly neighbouring Djibouti and Somalia and propagate the risk of spread to other regions. For each country, we proportionally sample 50 start locations, weighted by the estimated force of infection due to sylvatic spillover [11]. We then examine the risk for each of those 50 start locations through equation (2) ie. through examining the likelihood of moving from a start location to other districts (using the radiation model). Finally, we take the mean risk per district across all simulations as our estimate of the risk of introduction.

The code for the model used for the risk of introduction can be found at https://github.com/mrc-ide/YF_WHO_risk_reports.

### Risk of outbreak propagation

We define an outbreak as the occurrence of one or more human YF severe infections, where the severity spectrum follows Johansson et al. [12]. To model outbreak occurrence, we use a stochastic, dynamic model of YFV transmission which incorporates both sylvatic spillover and human-to-human (urban) transmission [11]. The key parameters for this model are the force of infection for sylvatic spillover, *λ*_*S*_, and the basic reproduction number of human-to-human transmission, *R*_0_. To estimate these parameters, Fraser et al. first assumed the force of infection and basic reproduction number per region were functions of environmental covariates including: a) *Aedes aegypti* occurrence, b) middle infrared reflectance, c) non-human primate species richness, d) Human population size, e) Temperature suitability index, and f) the type of land cover. They then utilised available epidemiological data on death notifications, case notifications and seroprevalence to estimate the model coefficients within a Bayesian framework. In this way, the force of infection and basic reproduction number may be projected in regions with only partial or no epidemiological YF data given the environmental conditions. Full details are presented in [11] and the median estimates of force of infection and basic reproduction numbers are shown in Additional file 1: Fig. S3.

In order to calculate the risk that an outbreak could spread or propagate given the introduction of YFV to a new region, we first sampled 1,000 sets of values of the basic reproduction number calculated for each 1st-level administrative region, using 1,000 sets of parameter values sampled from the posterior distribution estimated by Fraser et al. These values are constant over time and can be taken as a year-round average, as the model does not currently capture seasonality or other temporal variation in YFV transmission parameters.

For each set of basic reproduction number values, we introduce one infected individual to each region at the start of 2023 and simulate transmission over the following year using the dynamic model. 10 stochastic repetitions are carried out for each set of values. An outbreak is deemed to occur if there are one or more severe infections in the year after introduction when the number of severe infections is averaged over the stochastic repetitions. The relative risk of outbreak propagation is equal to the fraction of simulations where an outbreak is deemed to occur.

Note: we assume that there is no established sylvatic reservoir in these countries, and therefore the spillover force of infection is zero. We cannot say for certain that YFV is absent from non-human primate populations in these countries. However, as no sylvatic spillover events have been reported in these countries, and the species diversity is comparatively low, as collated by the International Union for Conservation of Nature (IUCN) redlist [13], we would expect this scenario to be a plausible, conservative assumption.

Furthermore, we include the risk of outbreak propagation for Yemen but note that Yemen is not included in the radiation model. Therefore we assume introduction occurs by flights or other means.

The code for the model of risk of outbreak propagation can be found at https://github.com/mrc-ide/DSY_YF_outbreak_risk2.

### Relative outbreak risk

To calculate the relative risk of an outbreak we multiplicatively combine the risk of potential spread of an outbreak given an infectious individual with the landscape of outbreak occurrence probability. In summary, the “risk of introduction" section provides a relative risk of introduction; the “risk of outbreak propagation" section provides an underlying probability that an introduced infectious individual will propagate an outbreak. The product of these two outputs provides a relative risk of outbreak conditional on an infection being introduced.

All analyses were conducted in R version 4.3.1 or later. Data was generated in R using shapefiles from GADM (version 3.6) https://gadm.org/index.html, and transferred to maps based on World Health Organization shapefiles. Maps were generated in R or, in the case of flowcharts/ diagrams, Microsoft Powerpoint.

## Results

### Risk of introduction

**Fig. 2:**
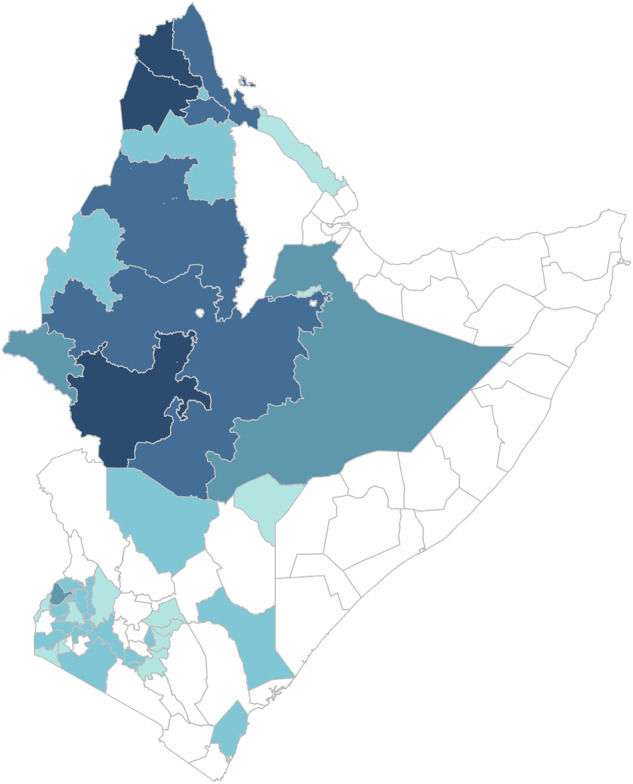
Start locations (1st administrative level) in neighbouring countries to Djibouti and Somalia (Eritrea, Ethiopia and Kenya). 50 samples were taken per country; a darker blue indicates that a district was sampled at a higher frequency

**Fig. 3:**
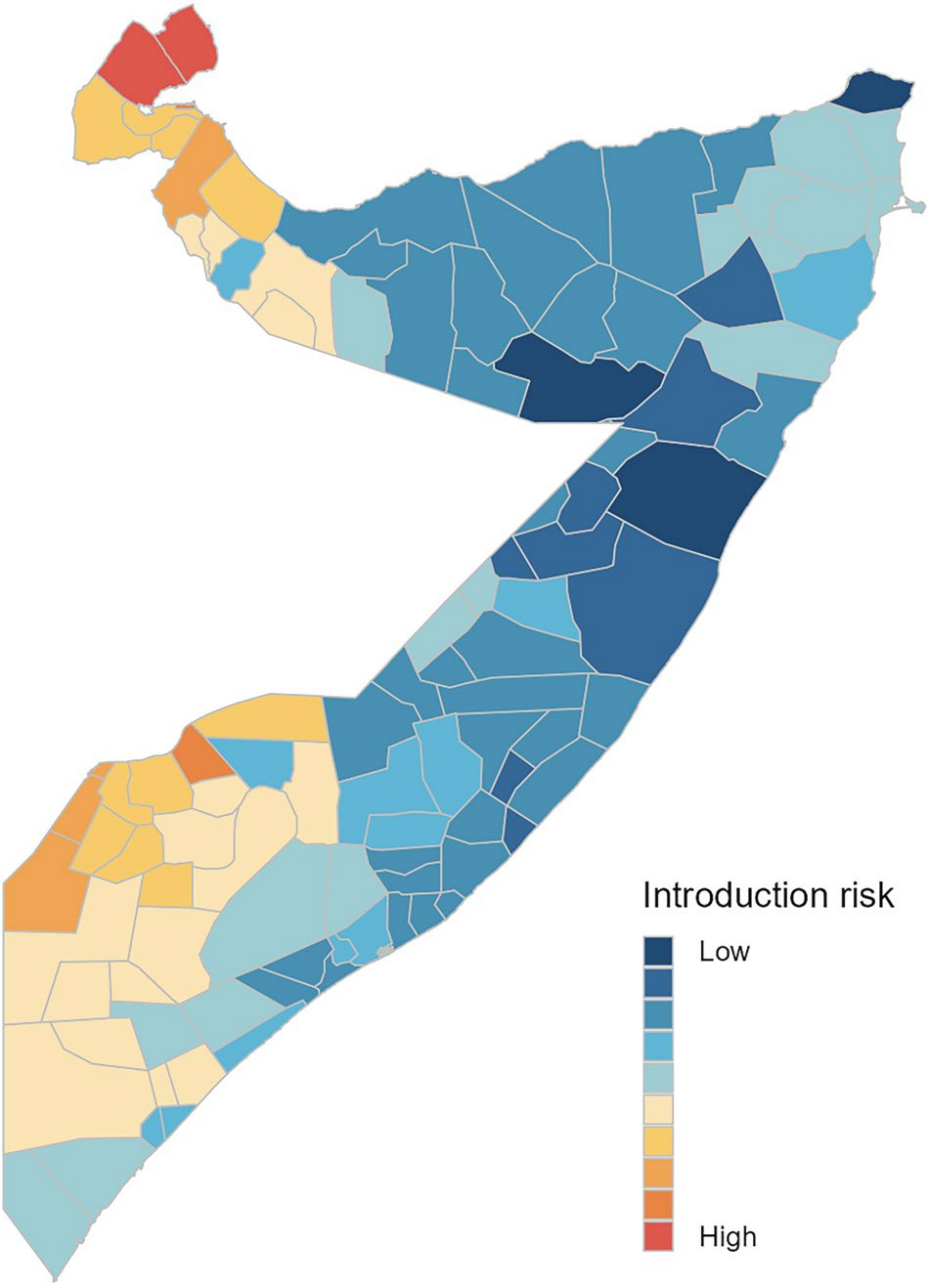
Relative risk of introduction of YF to 2nd-level administrative regions in Djibouti and Somalia

We first consider the risk that an infected individual travels from a YF endemic country to regions of Djibouti or Somalia. The fifty start locations per endemic country are shown in Fig. 2. Figure 3 shows the relative risk of introduction in regions of Djibouti and Somalia. The regions most at risk of YF importation are found in the west of Somalia and in Djibouti, with the highest risk regions being Tadjourah, Obock and Djibouti City in Djibouti and Rabdure in south-western Somalia. See Fig. 5 for final results when introduction risk is combined with risk of outbreak propagation.

### Risk of outbreak propagation

**Fig. 4:**
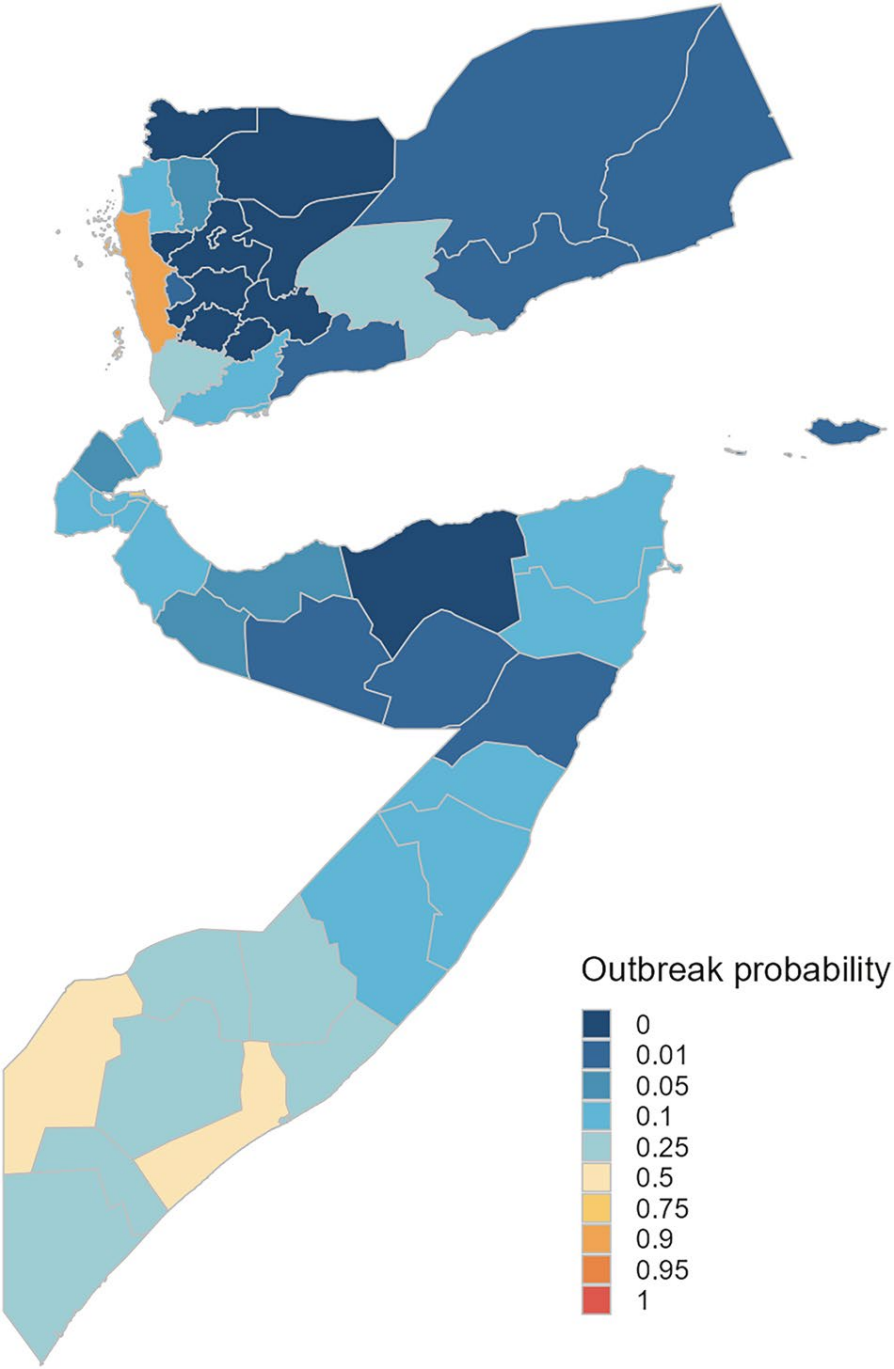
Map of the risk of outbreak propagation in first level subnational administrative regions of Djibouti, Somalia and Yemen in 2023, based on 1,000 simulations with 10 stochastic repetitions per simulation. The legend shows the proportion of simulations where an outbreak (defined as one or more severe infections) occurred

Figure 4 presents the probability of a single case propagating an outbreak. The highest risk (over 80%) is found in coastal western Yemen (Al Hudaidah) and Djibouti City. Other regions with a risk over 50% are found in southern Somalia (Gedo and Lower Shabelle).

Additional file 1: Fig. S5 shows the mean size of outbreaks across subnational administrative regions of Djibouti, Somalia and Yemen. This highlights that areas such as those in south west Somalia, Djibouti City and western Yemen could potentially see notable outbreaks if infections were introduced.

### Relative outbreak risk

**Fig. 5:**
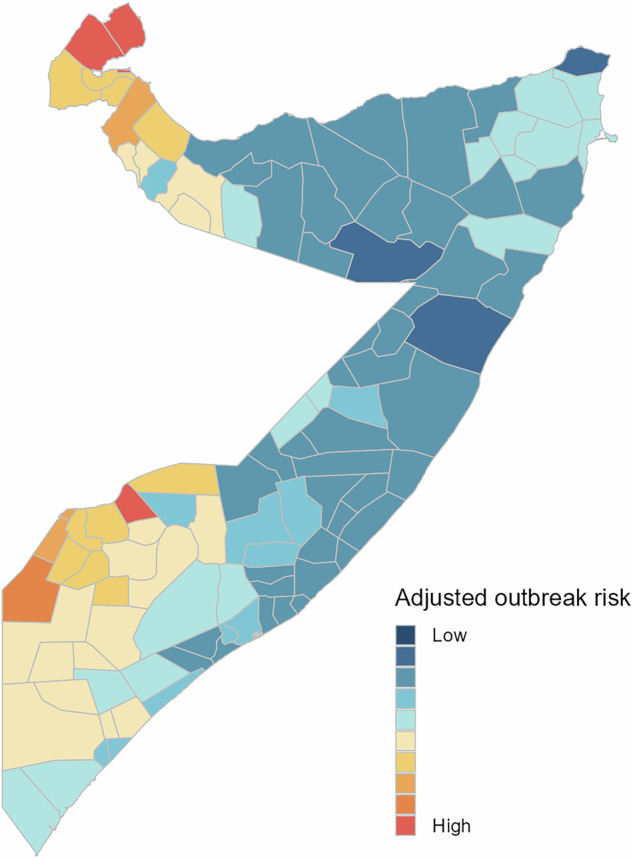
Map of relative outbreak risk in second level subnational administrative regions in Djibouti and Somalia in 2023 calculated via multiplying mean introduction risk scores for each region by the underlying outbreak risk based on a single introduced case for the corresponding first level subnational administrative region

Figure 5 presents the final relative risk of an outbreak in Djibouti and Somalia, based on mean introduction risk values and the risk of propagation. The risk is concentrated in border areas, particularly in the south-west and north-west.

## Discussion

We have examined the threat that YF poses to three countries considered at potential or moderate risk by the EYE Strategy: Djibouti, Somalia, and Yemen. We considered the risk that a case in neighbouring endemic countries could lead to the introduction of YFV through land-based movement, as well as the risk that the introduction of an infected individual could spark an outbreak given the environmental conditions. In Djibouti and Somalia, border areas are potentially vulnerable to YFV introduction through land-based movement, most notably in Djibouti city and south-west Somalia. When we consider the potential for an outbreak to propagate given introduction in Djibouti, Somalia, and Yemen we note that the same areas have potential for outbreak propagation but also highlight that a case introduced into Western Yemen has high potential to propagate an outbreak. Finally, when we couple the risk of introduction with the risk of outbreak propagation, we arrive at the conclusion that Western Djibouti and Southwestern Somalia are potentially vulnerable to YF outbreaks. This threat is due to geographic proximity and likely transport routes with endemic areas and the estimated environmental suitability for YFV transmission.

Our results highlight the risk of YF in a landscape already affected by 1) other arboviruses and 2) complex humanitarian crises which will complicate interventions [14]. Dengue is a notable issue in Yemen [15–17], Somalia [18] and Djibouti [19] and shares the same urban vector as YF. The Yemen conflict, which entered its tenth year in 2024, has led to malnutrition, health facility closures and increasing vulnerability to vaccine-preventable diseases [20]. This conflict may have also led to an increased number of reported cases of dengue [15]. A YF outbreak in these settings could present a huge challenge in terms of surveillance and response. Vaccination is the primary form of routine control for YF and the key emergency response in an outbreak, and global stockpiles were expanded in 2016 to hold six million doses for outbreak response [21], approximately 11% of the total population sizes for Djibouti, Somalia and Yemen in 2023 [22]. Our results highlight the need for further research to understand vector competence and cross-reactive immunity as well as the consideration of YF within testing and diagnosis strategies for compatible clinical presentations.

Other studies have raised the issue of YF in Djibouti, Somalia and Yemen through circulation of the virus in humans as well as presence of key YF vectors. In Djibouti, Andayi et al. estimated overall seroprevalence at 1.5% for YFV in 2010; however, whilst this could be due to natural infection, they suggest this may be attributable to vaccinated individuals [23]. The presence of *Aedes aegypti*, the urban vector of YFV, in Djibouti, Somalia and Yemen is well established [24]. There is also concern that the range of *Aedes*-borne viruses will increase with climate change, thus increasing the burden in newly at-risk countries [5, 6, 25]. Similarly human mobility, which has been highlighted as a risk factor for other arboviruses [26], is likely to increase with increasing climate pressures, leading to further potential for dissemination of outbreaks [27].

There are a number of modelling assumptions required to conduct these projections. The risk of introduction analysis assumes that human movement is land-based and motivated only by population sizes and distance between start and destination; however, we do not account for changes over time, by season, or alternative travel routes such as air and sea travel. As a result, we omit Yemen from this component despite its proximity to Eritrea, Djibouti, and Somalia. The risk of introduction also assumes movement occurs in line with the infectious window whereas it may be that longer journeys are less likely to be completed within the infectious period. Furthermore, we assume national borders are completely porous which may vary by region and country, and do not include a measure of yellow fever vaccination requirements at border crossings. Given these limitations/ assumptions our estimates of risk in border regions may be pessimisticand we will miss the fluctuation in risk due to time-varying migration and mobility. As more granular projections and data on cross-border and seasonal human movements become available, these estimates of risk may be refined.

When modelling the probability that an outbreak will propagate on introduction, we inherit the limitations of the model of Fraser et al. [11]. Particularly, this model is estimated from epidemiological data which is only available from some high-risk or endemic countries. As such, when we project the risk in Djibouti, Somalia and Yemen we utilise the estimated model’s relationship to the average environmental conditions rather than epidemiological data from those countries. Additionally, whilst the existence or endemicity of dengue in these countries is suggestive of a suitable transmission environment, it may imply there would be cross-reactivity with yellow fever, which is not accounted for in this analysis as we assume populations are fully susceptible. We also assume that there is no existing sylvatic reservoir in the countries studied; however, we cannot be certain of this as there is little surveillance of non-human primates. Thus, we may assume the projections of the risk of outbreak propagation are highly uncertain.

## Conclusions

Countries and regions bordering existing YF endemic regions are potentially vulnerable to both introduction of YF cases and subsequent outbreak spread. Changing patterns of human movement and displacement, as well as changing environmental conditions through climate change, could exacerbate this risk. We highlight and quantify areas of relatively higher risk in Djibouti, Somalia and Yemen. This complements ongoing work from the WHO EYE Strategy, and its post 2026 transition toward a broader Global Arbovirus Strategy, and further emphasises the importance of awareness of YFV importation risk when considering clinical surveillance.

## Supplementary information

Supplementary material 1

## Data Availability

Data and R code used in this work is available in the two GitHub repositories below:- Risk of introduction: https://github.com/mrc-ide/YF_WHO_risk_reports- Risk of outbreak propagation: https://github.com/mrc-ide/DSY_YF_outbreak_risk2The risk of introduction is also available to explore as an online application, RAPTORX, https://shiny.dide.ic.ac.uk/raptorx/. This methodology was initially developed for examining the potential spread of YFV in the outbreaks of 2016 in the Republic of Angola and the Democratic Republic of the Congo and has since been further refined.

## List of abbreviations

WHO: World Health Organization
EMRO: Eastern Mediterranean Regional Office
EYE strategy: Eliminate Yellow fever Epidemics strategy
YF: Yellow fever
YFV: Yellow fever virus
IUCN: International Union for Conservation of Nature
