# Supplementary material for: Yellow fever outbreak potential in Djibouti, Somalia and Yemen: a mathematical modelling study
Source: BMC Glob Public Health. 2026 Jun 8;4:57. doi: 10.1186/s44263-026-00284-9 (PMC13245028; doi:10.1186/s44263-026-00284-9)
Supplement: Supplementary file 1 — Supplementary material 1 [file 44263_2026_284_MOESM1_ESM.pdf]

Yellow fever outbreak potential in Djibouti, Somalia and Yemen: a mathematical modelling study - Additional file 1

Keith Fraser[1], Laurence Cibrelus[2], Jennifer Horton[2], Chiori Kodama[2], J Erin Staples[3], Katy A M Gaythorpe[1*]

[1*]MRC Centre for Global Infectious Disease Analysis, School of Public Health, Imperial College London, Wood Lane, London, United Kingdom.
[2] World Health Organisation, Avenue Appia 20, 1211 Geneva, Switzerland.
[3]Division of Vector-borne Diseases, Centers for Disease Control and Prevention, Fort Collins, Colorado, United States.

*Corresponding author(s). E-mail(s): k.gaythorpe@imperial.ac.uk;

S1 EYE strategy risk categorisations, 2016

Risk categories according to the EYE Strategy in 2016 [1]. Djibouti is categorised as potential risk, and Somalia is categorised as moderate risk.

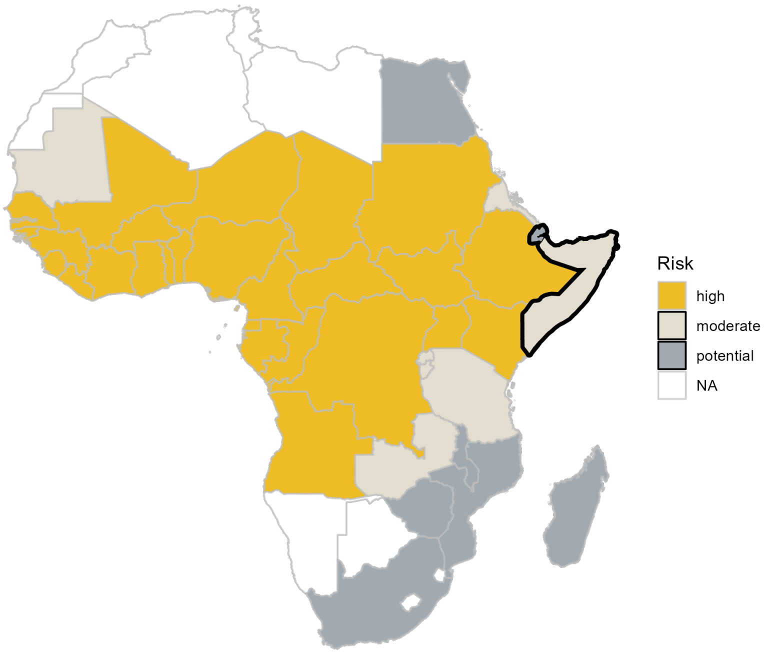

Fig. S1: Adapted visualisation of EYE strategy risk categories in Africa, 2016 [1]. High risk countries are shown in yellow, moderate risk in beige, potential risk in grey and unspecified risk in white. Djibouti and Somalia are outlined in black.

S2 Dynamic model of yellow fever transmission from Fraser et al. 2024

The dynamic model of yellow fever transmission used in this work to simulate the effects of YF introduction to the regions of interest is a compartmental model described in full in the supplementary material of Fraser et al [2]. Figure S2 shows a simplified diagram of the transmission model. The vaccinated (V) compartment is omitted since we do not include vaccination in this work due to considering non-endemic regions where significant vaccination is assumed not to currently be carried out.

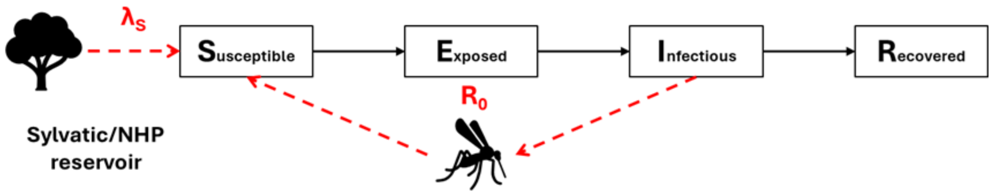

Fig. S2: Diagram of transmission model

The key equations of the model are given below. The susceptible (S), exposed (E), infectious (I) and recovered (R)populations are stratified by age (A) into groups 1 year apart, with P being the total population across all age groups. The total force of infection λ_{TOT} is calculated using equation (1), with the number of new infections per unit time by age group $E_{NEW}(A)$ calculated using a binomial expression (equation (2)). Population sizes are then calculated as shown in equations (3).

$$\lambda_{TOT} = \lambda_S + \frac{R_0}{t_{INF}} \frac{\sum(I)}{P} \tag{1}$$

$$E_{NEW}(A) = \binom{S(A)}{\lambda_{TOT}} \tag{2}$$

$$\begin{aligned}
\frac{dS(A)}{dt} &= -E_{NEW}(A) + \Delta P_S(A) \\
\frac{dE(A)}{dt} &= E_{NEW}(A) - \frac{E(A)}{t_{INC} + t_L} \\
\frac{dI(A)}{dt} &= \frac{E(A)}{t_{INC} + t_L} - \frac{I(A)}{t_{INF}} \\
\frac{dR(A)}{dt} &= \frac{I(A)}{t_{INF}} + \Delta P_R(A)
\end{aligned} \tag{3}$$

λ_S is the additional force of infection due to sylvatic spillover. This was set to zero in this work due to the assumption of a lack of a sylvatic reservoir. The model includes a vaccination component [2], but this is omitted here (with the relevant parameters set to zero) due to the assumption that no vaccination is carried out in the relevant regions. $\Delta P_S(A)$ and $\Delta P_R(A)$ are population adjustments in the susceptible and recovered groups due to demographic change; these were calculated based on United Nations World Population Prospects data [3]. t_{INF}, t_{INC} and t_L are the infectious, incubation and latent periods respectively (all set to 5 days).

S3 Force of infection and basic reproduction number estimates from Fraser et al. 2024

Figure S3 shows how median values of the sylvatic spillover force of infection and basic reproduction number, R_0, are expected to vary across the three countries considered based on environmental covariate values [2]. Note that we only use R_0 in this work as the sylvatic spillover force of infection is tied to a sylvatic reservoir which is assumed not to be present, as discussed in the methods for risk of outbreak propagation. Figure S4 shows the distribution of R_0 values for each region.

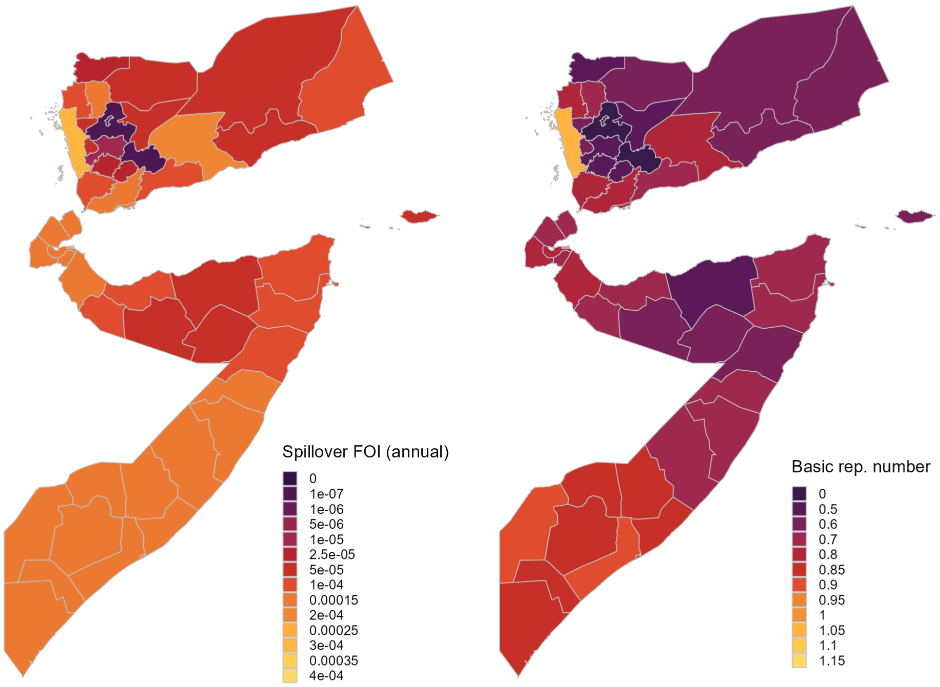

(a) Force of infection

(b) Basic reproduction number

Fig. S3: Median values of (a) annual force of infection for sylvatic spillover and (b) basic reproduction number for human-to-human transmission in first-level subnational administrative regions of Djibouti, Somalia and Yemen, calculated from environmental covariates.

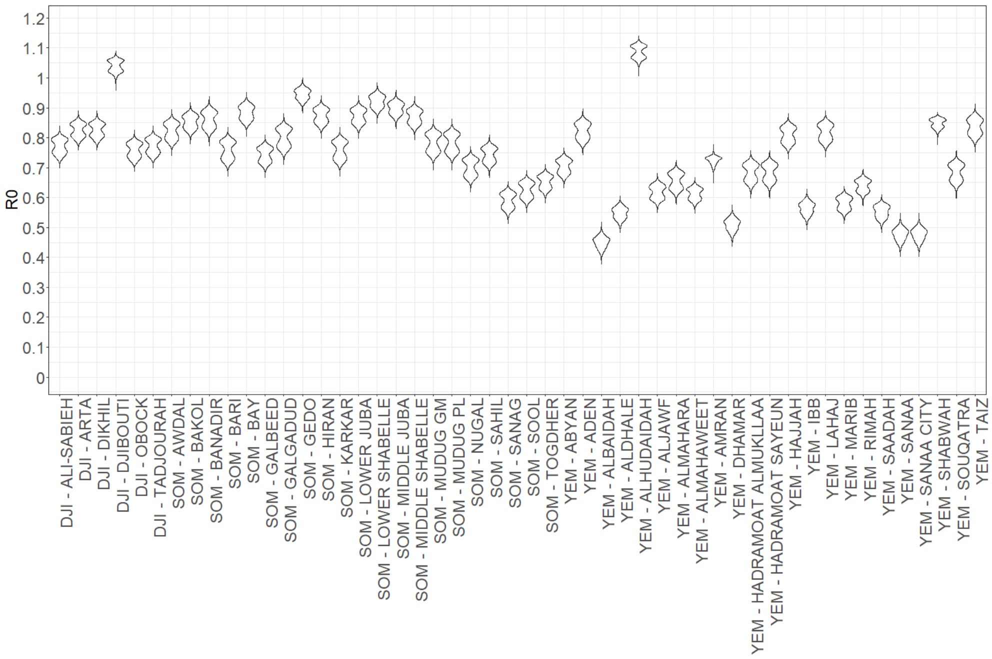

Fig. S4: Violin plot of R_0 values by region

S4 Outbreak size

Figure S5 below shows the mean size of outbreaks across 1st-level subnational administrative regions of Djibouti, Somalia and Yemen. Outbreak size is measured in number of severe infections, and so has a value of at least 1 when an outbreak is recorded under the definition given in the ”risk of outbreak propagation” method section. Regions where the simulated outbreak risk is zero therefore have a mean outbreak size of zero (dark blue). The regions of high mean outbreak size correspond to the regions of high outbreak risk (see Figure 3 in the main text).

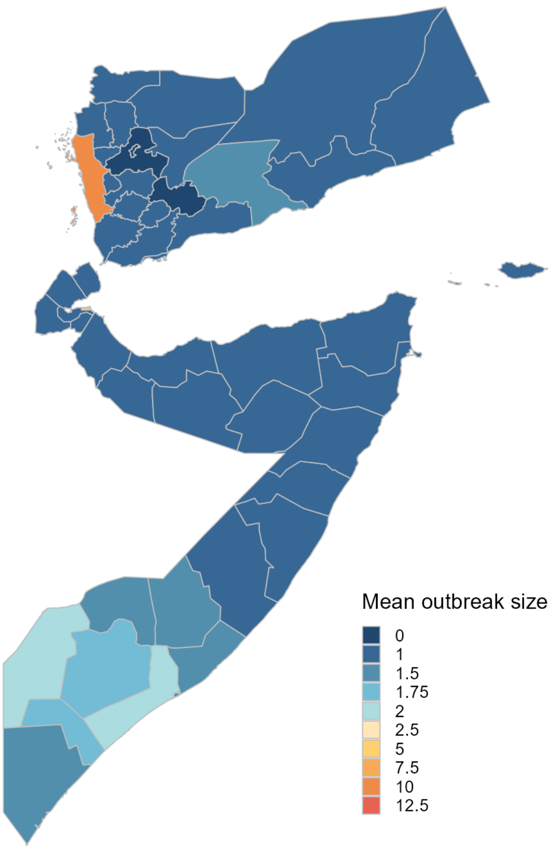

Fig. S5: Map of mean outbreak size (measured in number of severe infections) in first-level subnational administrative regions of Djibouti, Somalia and Yemen, calculated from simulated infection data used to calculate outbreak risk

References

[1] World Health Organization: Eliminate Yellow Fever Epidemics (EYE) 2017 – 2026 (2018). https://www.who.int/publications/i/item/9789241513661

[2] Fraser, K.J., Hamlet, A., Jean, K., Ramos, D.G., Romano, A.P.M., Horton, J., Cibrelus, L., Ferguson, N.M., AM Gaythorpe, K.: Assessing yellow fever outbreak potential and implications for vaccine strategy. PLOS Global Public Health (2024)

[3] World population prospects. Technical report (2019). https://population.un.org/wpp/
